# Initial Experience With SARS-CoV-2-Neutralizing Monoclonal Antibodies in Kidney or Combined Kidney-Pancreas Transplant Recipients

**DOI:** 10.3389/ti.2022.10109

**Published:** 2022-03-31

**Authors:** Friederike Bachmann, Klemens Budde, Norbert Suttorp, Tilman Lingscheid, Miriam Songa Stegemann, Bilgin Osmanodja, Eva Schrezenmeier, Wiebke Duettmann, Ulrike Weber, Marcel Naik, Lukas Johannes Lehner, Andreas Kahl, Michael Duerr, Kai-Uwe Eckardt, Johannes Waiser, Mira Choi, Fabian Halleck

**Affiliations:** ^1^ Department of Nephrology and Medical Intensive Care, Charité-Universitätsmedizin Berlin, Corporate Member of Freie Universität Berlin and Humboldt-Universität zu Berlin, Berlin, Germany; ^2^ Department of Infectious Diseases and Respiratory Medicine, Charité-Universitätsmedizin Berlin, Corporate Member of Freie Universität Berlin and Humboldt-Universität zu Berlin, Berlin, Germany; ^3^ Clinician Scientist Program, Berlin Institute of Health at Charité—Universitätsmedizin Berlin, BIH Academy, Charité-Universitätsmedizin Berlin, Corporate Member of Freie Universität Berlin and Humboldt-Universität zu Berlin, Berlin, Germany

**Keywords:** SARS-CoV-2, kidney transplantation, immunosuppression, bamlanivimab, monoclonal antibodies

## Abstract

**Background:** Antiviral drugs have shown little impact in patient infected with acute respiratory coronavirus 2 (SARS-CoV-2). Especially for immunocompromised persons positive for SARS-CoV-2, novel treatments are warranted. Recently, the U.S. FDA has granted an emergency use authorization (EUA) to two monoclonal antibodies (mAb) targeting the viral spike protein: bamlanivimab and casivirimab and imdevimab. As per the EUA, all SARS-CoV-2 positive organ transplant recipients can receive mAb treatment.

**Patients and methods:** We queried our center’s transplant registry to identify SARS-CoV-2 infected recipients treated with single doses of either Bamlanivimab or casivirimab/imdevimab up to May 31, 2021. We analyzed clinical outcomes, renal function and virus-specific antibodies. The co-primary endpoints were hospitalization due to COVID-19 and SARS-CoV-2 RT-PCR negativity.

**Results:** Thirteen patients at a median interval of 55 (IQR, 26-110) months from transplant were treated: 8 with bamlanivimab and 5 with casivirimab/imdevimab. In all, 4/13 (31%) patients were hospitalized at some time, while 11/13 (85%) achieved PCR negativity. 2/4 hospitalized patients received mAb as rescue treatment. Overall mortality was 23%, with one death attributable to transplant-associated lymphoma. All six patients infected with the B 1.1.7 variant were alive at last contact. *Conclusion*: mAb treatment appears effective when administered early to SARS-CoV-2-infected transplant recipients.

## Graphical Abstract

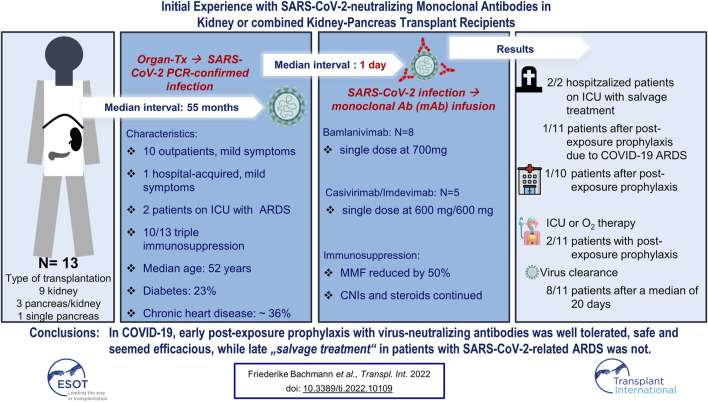

## Background

Until now, antiviral drugs have largely failed to improve the natural course of coronavirus disease 19 (COVID-19) (1). In fact, mainly dexamethasone had positive effects on patients severely affected by SARS-CoV-2 (2,3). Therefore, antibody treatments (4,5) attracted strong interest prior to the availability of vaccines in order to ameliorate disease severity in subjects with proven infection. In addition to convalescent plasma (6), also monoclonal and “off-the-shelf” preparations of neutralizing intravenous immunoglobulins may be effective in fighting SARS-CoV-2 infection (7). Convalescent plasma might positively impact intensive care unit (ICU) admission and mortality rates (6). However, these preparations are difficult to standardize and a drug containing high amounts of a well-defined epitope-specific antibody might be superior and exert more reliable efficacy. In fact, two compounds have been granted an emergency use authorization (EUA) by the U.S. FDA for mild to moderate COVID-19 since November 2020 (8,9). In Germany both compounds are provided for unlicensed use since January 2021 due to pending approval in Europe.

Bamlanivimab, one of these two compounds, constitutes a neutralizing IgG1 monoclonal antibody (mAb) binding to the receptor binding domain (RBD) of the spike protein of SARS-CoV-2 (9). Bamlanivimab was shown to accelerate elimination of SARS-CoV-2 and, more importantly, reduced probability of emergency department visits by approximately 75% when given to outpatients (7). Casivirimab and imdevimab are two neutralizing mAbs targeting two different epitopes of the RBD (10). They are administered together as an “antibody cocktail” with a single dose in subjects who are at high risk for developing severe COVID-19 (5,11). The combined phase 1–3 trial met its key efficacy endpoints, in that viral load over time was significantly reduced and medically attended visits were less frequent in patients receiving any dose of casivirimab/imdevimab (11).

Chronic kidney disease (CKD) is associated with a higher risk of in-hospital mortality in COVID-19 patients than, for instance, malignancy or cardiac comorbidities, as demonstrated in several large studies (12,13). Overall mortality in hospitalized CKD patients was 26% (12). While many CKD patients are of advanced age with age being another major risk factor for poor outcomes, kidney transplantation constitutes a further risk factor for SARS-CoV-2 infected individuals (14,15). Besides immunosuppression, graft function was inversely correlated with COVID-19 disease severity (16). Because immunosuppression may suppress adequate production of protective antibodies, the use of external antibody preparations may be of higher effectivity in kidney transplant recipients, known to be at high risk for poor outcomes. Until now, experience with neutralizing mAbs in COVID-19 kidney transplant recipients under a defined immunosuppression is very limited. In a recent single-center, retrospective study, bamlanivimab reduced the hospitalization rate in a cohort where approximately 30% of patients were of immunosuppression. Of note, the authors estimated the number needed to treat (in order to prevent one hospitalization) to be 8 (17). To the best of our knowledge, only one case series from outside the U.S. (18). has been reported so far.

## Patients and Methods

We queried our electronic patient database “TBase” (19) for the search terms “SARS-CoV-2,” “COVID-19” and “bamlanivimab” or “casivirimab” or “imdevimab.” Retrieved records were manually reviewed for all patients receiving treatment with mAbs preparation “bamlanivimab” or “casivirimab/imdevimab” until May 31, 2021. All patients had a minimum follow-up of 14 days after mAb infusion, the last of which was administered on May 22, 2021.

According to indication, solid organ transplant (SOT) recipients with a positive SARS-CoV-2 PCR were treated with 700 mg bamlanivimab or 1200 mg casivirimab/imdevimab. The decision to treat was made by a multidisciplinary team consisting of nephrologists, transplant physicians and infectious disease specialists. We collected clinical outcomes, medication history, laboratory results for infection parameters, renal function tests, blood count, and anti-SARS-CoV-2 serum antibodies (IgA and IgG using Euroimmun–ELISA, Lübeck, Germany). All patient samples from the current series were analyzed using NGS-based sequencing of viral genomes as described previously (20). Methods differed based on viral load in patients’ nasal swab. Briefly, libraries were established using the KAPA RNA Hyper Prep kit (high viral load) or a PCR amplicon-based sequencing approach.

Imaging results were used to assess pulmonary infiltration patterns in patients with dyspnea. Telemedicine support, which has been in use for our KTR recipients for about 1 year, was offered to all patients following mAb treatment and comprised symptom-reporting, remote vital sign monitoring, medication intake and chat functioning (21). The co-primary endpoints were hospitalization due to COVID-19 by day 29 (11) and viral response, respectively. The latter was defined as a SARS-CoV-2 RT-PCR negative (swab) test on day 11 (+/− 2 days) after mAb administration. Sustained response was defined as an ongoing negative PCR negativity at further testing. In contrast to mAb pivotal studies, we used dichotomized PCR test results rather than log reduction of viral load. We additionally defined a composite secondary endpoint which was defined by admission to ICU, any form of ventilation, or death. Disease-specific survival was calculated from first positive SARS-CoV-2 PCR test until death from any cause or last follow-up. Cause of death for deceased patients was attributed to COVID-19 if their last PCR was positive and/or they met criteria for severe COVID-19 at last follow-up and/or they died due to pulmonary involvement of a previously documented SARS-CoV-2 infection.

## Results

In total, thirteen organ transplant recipients were identified and included in this report. The median age was 52 (IQR 42-60) years. Baseline demographics are shown in Tables 1, 2. Patients were diagnosed with SARS-CoV-2 infection at a median of 55 months (IQR 26-110 months) after organ transplantation. None of the patients was fully vaccinated when testing positive. In six of all sequenced samples (46.1% of all patients), the B.1.1.7 lineage of SARS-CoV-2 virus was confirmed at baseline. At the time of diagnosis, 10 (76.9%) patients received triple immunosuppression consisting of a calcineurin inhibitor (CNI; tacrolimus or cyclosporine A), mycophenolic acid (MPA), and corticosteroids. Three (23.1%) patients were on a steroid-free regimen. Three patients were diabetic, and four patients had chronic cardiac disease. Four (30.7%) patients received telemedical care. Median interval from diagnosis confirmed by positive SARS-CoV-2 PCR test to administration of either mAb treatment was 1 day (range, 1–30) and median follow-up 40 days (IQR 23–50 days) (Table 1). Bamlanivimab was administered to the first eight patients, including all three hospitalized patients. When casivirimab/imdevimab became available at our center, we switched mAb treatment to this compound. Both antibody preparations were well tolerated and we did not observe any infusion-related reactions.

**TABLE 1:** Patients’ characteristics.

Variable	
Median age, years (IQR)	52 (42–60)
Male gender, N (%)	8 (61.5)
Kidney transplant, N (%)	9 (69.23.6)
Kidney-pancreas; single pancreas transplant, N (%)	3(23.1); 1(7.7)
Living donor, N (%)	5 (38.5)
BMI, median (range)	23 (16–32)
Diabetes, N (%)	3 (23.1)
Chronic heart disease, N (%)	4 (36.3)
Median interval SOT to SARS-CoV-2 positivity, months (range)	55 (26–125)
C-reactive protein (mg/L), median (range) at diagnosis	7 (2–106)
Lactate dehydrogenase (U/l), median (range) at diagnosis	246 (192–511)
Serum interleukin-6 (pg/ml), median (range) at diagnosis	14 (1.6–80.6)
Interval positive PCR—administration of MoAb, days (range)	1 (1–30)
Oxygen supplementation, N (%)	3 (23.1)
ICU admission, N (%)	3 (23.1)
COVID-19 symptoms at treatment: moderate/severe, N (%)	3 (23.1)
Median duration of follow-up, days (IQR)	40 (23–50)
Patients alive at last follow-up, N (%)	10 (76.9)
Renal replacement therapy, N (%)	3 (15.4)

**TABLE 2:** Outcomes.

**Pat. No.**	**Gender**	**Donor Type**	**Immuno-suppression at COVID-19 dx**	**Interval transplant- SARS-CoV-2 positivity PCR (months)**	**Interval diagnosis to mAB treatment (days)**	**Variant of concern**	**Anti-SARS-CoV-2 S1 IgG/IgA antibodies baseline/course of the disease**	**Oxygen supplementation due to COVID-19**	**ICU admission/ventilation type due to COVID-19**	**Response/Sustained viral response**	**Survival status**
1	M	Deceased donor	Tac, MMF, steroid	171	2 days	no	Positive on day 14 after diagnosis	yes	Yes; mechanical ventilation	y/n	Deceased on day 57 after Dx^b^
2	M	Deceased donor	Tac, MMF, steroid	45	1 day	no	Positive on day 83 after diagnosis	no	No	y/y	Alive, stable renal function
3	M	AB0i kidney	Tac, MMF	64	1 day	B.1.1.7	Positive on day 21 after diagnosis	no	no	y	Alive, stable renal function
4	F	Deceased donor	Tac, MMF	55	2 days	B.1.1.7	Positive on day 88	no	no	y	Alive, stable renal function
5	M	Kidney-pancreas	CyA, SRL, MMF	276	2 days	B.1.1.7	Positive on day 109 after diagnosis	no	no	y	Alive, stable renal function
6	F	Kidney-pancreas	CyA, MMF, Pred	239	1 day	no	Positive on day 105 after diagnosis	no	no	y	Alive, stable renal function
7	F	Kidney-pancreas	CyA, MMF, steroid	15	1 day	no	Positive on day 43	no	no	y/n.a.	Alive, stable renal function
8	F	Living donor	Tac, MMF, steroid	1	1 day	B.1.1.7	Positive on day 14 after Dx	no	no	y/y	Alive, stable renal function
9	M	AB0i kidney	Tac, MMF, steroid	18	1 day	B.1.1.7	Positive on day 88	no	no	y/n.a.	Alive, stable renal function
10	F	AB0i kidney	Tac, MMF, steroid	45	1 day	B.1.1.7	Pos on day 14 after Dx	no	No	y/y	Alive, stable renal function
11	M	Single pancreas	Tac, MMF, steroid	59	Nosocomial infection 1day	no	Negative on day 14, positive on day 78	no	no	y/y	Alive, discharged^a^ on day 54 after Dx, stable renal/pancreas function
12^d^	M	Deceased donor	Tac, MMF, steroid	26	15 days	no	Pos on day 13	yes	Yes; mechanical ventilation	no/no	Deceased on day 45
13^d^	M	Living donor	Tac, steroid	82	30 days	no	Pos on day 30	no	no	no/no	deceased on day 60 due to progressive PTLD^c^

Abbreviations: COVID, Coronavirusdisease 2019; mAb, monoclonal antibody; M, male; F, female; Tac, tacrolimus; CyA, cyclosporine A; MMF, mycophenolic acid; Dx, diagnosis; n.d., not done; IgA, Immunglobulin A; IgG, Immunglobulin G; S1, Spike antigen; ICU, intensive care uni; PCR, polymerase chain reaction.

a Prolonged inpatient care due to arterial occlusion.

b Became negative on day 19 and turned positive on day 27.

c Duration positivity 60 days until death.

d mAB, were administered as rescue therapy.

For two of three patients (Table 2: patients 12 and 13) who were already hospitalized and had received other treatments, including dexamethasone and convalescent plasma, mAb administration was delivered as a rescue treatment. Therefore, MMF was discontinued and tacrolimus dosage was adjusted to trough levels of 4–6 ng/ml. One of these two patients had refractory PTLD/acute lymphoblastic leukemia with leukemic meningitis. Both patients had acute kidney injury requiring dialysis. They died on day 45 and day 60, respectively, after diagnosis of intractable COVID-19 with virus persistence until death.

Another hospitalized patient (Table 2: patient 11) developed nosocomial COVID-19 infection during treatment of peripheral vascular disease. MMF was discontinued. He was treated in-line with EUA on day 1 after diagnosis with moderate symptoms. RT PCR became negative on day 24 after diagnosis with his further clinical course being dominated by vascular disease-associated complications. Renal function was stable (on day 54 after diagnosis) and remained unaltered after discharge.

All remaining 10 patients received mABs during outpatient care with mild symptoms and according to the respective notice of the general ruling by the Federal Ministry of Health (Bundesgesundheitsministerium, BMG). Of note, none of the six carriers of the B.1.1.7 variant needed inpatient care or oxygen supplementation. In addition to standard PCR tests, six patients were tested for the presence of pre-infusion anti-SARS-CoV-2 antibodies. All six were seronegative. As patients were not treated within a prospective study, testing for serum antibodies was performed at discretion of the treating physician in charge.

Therefore, previous infection and preexisting immunity seems unlikely. MMF was reduced by 50% and steroids continued in patients on a steroid maintenance regimen. Despite continued immunosuppression following their organ transplants, 9/10 had an uneventful outpatient course and did not develop severe COVID-19 symptoms requiring hospitalization. SARS-CoV-2 PCR became negative in 6 patients after a median of 22 days (range 18–35). However, one patient with initial viral clearance had symptomatic disease recurrence, and multiple positive PCR tests later on. He was hospitalized on day 21 after diagnosis and died due to COVID-19 associated ARDS in the ICU on day 57 after initial disease onset.

In summary, two out of 11 patients (18%) with early antibody treatment reached the composite endpoint and were admitted to an ICU and required oxygen. With one patient dying due to Covid-19, mortality was still app. 10% in our series. This underscores an ongoing medical need in the severely immunocompromised. In this cohort, time to viral clearance occurred after a median of 20 days, and sustained viral clearance was achieved in 8 patients (73%). Testing for serum antibodies against one subunit of the spike protein (anti-S1 Ig A and IgG) revealed positive results for anti-S1 IgG in all patients after a median of 43 (range, 13–109) days.

## Discussion

Immunocompromised subjects with SARS-CoV-2 infection are prone to an unfavorable course of the disease with a 10-fold increased mortality risk. Pre-emptive administration of monoclonal, virus-neutralizing antibodies that have constant, defined and reproducible characteristics has shown to benefit subjects with mild symptoms from confirmed SARS-CoV-2 infection (7, 10 and 12). We treated 13 consecutive SOT recipients from our center. All but one patient who had received pre-emptive treatment with the mAbs are alive after a follow-up of 40 days. Only one of these 11 patients experienced recurrence of viral infection and eventually died from intractable COVID-19. This patient’s fate leads to the speculation that, in light of the half-life of the mAbs (13 days for bamlanivimab and 13–18 days for casivirimab/indevimab) (22), a single dose might not be appropriate in severely immunocompromised patients or viral immune escape took place due to mAb-monotherapy and insufficient immune-response by the patient. Two severely ill patients received bamlanivimab at a relatively long interval from SARS-CoV-2 infection and died due to complications from underlying disease and refractory COVID-19. Findings for anti-S1 IgG showed positive results for all patients after a median of 43 days from mAb administrations. Especially positive results after app. 80 days from therapeutical antibody infusion suggest “true seroconversion” rather than remaining concentrations of bamlanivimab or casivirimab/imdevimab, respectively. The overall good outcome is particularly remarkable, since in 6 of 13 cases the B.1.1.7 lineage was found to be the infectious agent, which is associated with higher reproducibility and case fatality. All 6 subjects carrying the B.1.1.7 lineage had an uneventful course without need for oxygen or other interventions, suggest efficacy against this variant of concern. Another strength of our study is the fact that prior infection was ruled out for six patients assuming none of these having “natural” immunity. Our study has some limitations: first, the sample size is small. Second, in this exploratory pilot study we did not attempt comparison with a control group not receiving a monoclonal antibody. Third, allocation to any one of the mAbs was by availability and individual decision rather than randomization making any comparison impossible. The dynamic situation of the pandemic is mirrored by incoming virus variants which may escape from treatments established in earlier phases of the pandemic. Of note, early data of a novel compound, sotrovimab, indicate its efficacy also for the Omicron variant (23). Quickly evolving virus variants may pose a novel threat to communities by questioning established strategies in intervals as short as weeks. For instance, the Omicron type features a magnitude of mutations clustering in the receptor binding motif (RBM) leading to an immune escape not only to the first class of mAbs, but also to covalescent plasma and certain types of vaccinations. Interestingly, the non-RBM targeting second class of mAbs is still effectively neutralizing the Omicron variant as very recently shown by a Swiss group (24).

## Conclusion

Our initial experience with neutralizing mABs for SOT recipients with confirmed SARS-CoV-2 infection shows excellent tolerability and suggests high efficacy including infections with the B.1.1.7 variant. We conclude that in a setting of rescue therapy no clear benefit can be documented, a finding which is in accordance with FDA emergency use authorization while early administration appears efficacious in prevention of severe COVID-19 in heavily immunosuppressed patients with mild symptoms. However, rates of overall and sustained PCR responses were low, suggesting a potential discordance between viral replication and clinical course and the need for continued surveillance.

## Data Availability

The raw data supporting the conclusion of this article will be made available by the authors, without undue reservation.
